# Resource Allocation and Computation Offloading for Wireless Powered Mobile Edge Computing

**DOI:** 10.3390/s22166002

**Published:** 2022-08-11

**Authors:** Jun Chen, Zheng Chang, Wenlong Guo, Xijuan Guo

**Affiliations:** 1 The Key Laboratory for Computer Virtual Technology and System Integration of Hebei Province, Colleage of Information Science and Engineering, Yanshan University, Qinhuangdao 066004, China; 2 School of Computer Science and Engineering, University of Electronic Science and Technology of China, Chengdu 610056, China; 3 Faculty of Information Technology, University of Jyväskylä, P.O. Box 35, 40014 Jyväskylä, Finland

**Keywords:** mobile edge computing, offloading, wireless power transfer, full-duplex, half-duplex

## Abstract

In this paper, we investigate a resource allocation and computation offloading problem in a heterogeneous mobile edge computing (MEC) system. In the considered system, a wireless power transfer (WPT) base station (BS) with an MEC sever is able to deliver wireless energy to the mobile devices (MDs), and the MDs can utilize the harvested energy for local computing or task offloading to the WPT BS or a Macro BS (MBS) with a stronger computing server. In particular, we consider that the WPT BS can utilize full- or half-duplex wireless energy transmission mode to empower the MDs. The aim of this work focuses on optimizing the offloading decision, full/half-duplex energy harvesting mode and energy harvesting (EH) time allocation with the objective of minimizing the energy consumption of the MDs. As the formulate problem has a non-convex mixed integer programming structure, we use the quadratically constrained quadratic program (QCQP) and semi-definite relaxation (SDR) methods to solve it. The simulation results demonstrate the effectiveness of the proposed scheme.

## 1. Introduction

It is expected that future wireless networks can not only provide data and voice services to a massive number of MDs but also bring computational and artificial intelligence (AI) capabilities to the MDs. However, due to the size limitation, the MDs are typically constrained by computing capacity, storage capacity and battery capacity, which will prevent MDs from performing seamless computational tasks. Therefore, tackling the energy and battery capacity challenges urgently demand for developing new wireless network paradigms [1].

With the development of Internet technology and mobile network, the data generated by MDs and application have significantly increased [1]. In addition, the increasing demand for computing and data service from massive MDs have urged the development of a wireless network, which integrates the computing capability into the wireless network. Cloud computing emerges as a new computing paradigm which provides computing services to a large amount of MDs. The cloud computing server has powerful computing processing capacity, and MDs can offload computing tasks to the cloud server for computing processing. However, the cloud computing servers are usually distant from the MDs [2]. Therefore, during the process of task transmission, a large delay and a large amount of energy consumption will be induced, which is unfavorable to solving real-time task processing with the low time delay requirement of MDs.

In order to provide MDs with more proximity, lower latency and reduced energy consumption for computing services, MEC has been attracted considerable attention. MEC has the potential to provide computing capabilities close to the MDs within the radio access network, aiming to reduce transmission delay [3,4], improve network operational efficiency, and promote the service experience. In particular, MEC can be thought of as a cloud computing server running on the network edge to perform specific tasks that traditional network infrastructure cannot provide [5]. The applications or intensive computation tasks can be offloaded in a dynamic or statical mode to the resource-rich edge servers for execution, which helps reduce the transmission delay and decrease energy consumption so as to improve the quality of service [6,7] in a better way. For the partitioning of user tasks, it is possible to offload computational processing in binary and partial task divisions. In MEC systems, a large amount of existing works focus on optimizing offloading process based on the system energy consumption, time delay, and transmission throughput. It is also worth noticing that the MEC server can allocate computing resources to user tasks to improve the energy efficiency and reduce the overall energy consumption of the system [8].

Meanwhile, how to provide sustainable and efficient power supply for MDs to execute long-term task processing has also become a challenge in the current MEC network system. Due to the limitation of battery capacity, it is difficult or even impossible for MDs to maintain the processing of computing tasks for a long time. In order to solve the issue of energy supply, wireless energy harvesting is a promising technology which has been widely developed in MEC systems. In general, EH plays a crucial role in addressing the issue of battery capacity constraint and furthermore to support MDs to realize green energy-oriented and sustainable computing offloading services [9]. MDs with EH technology are capable of harvesting green energy from solar panels, wind and wireless energy sources for task offloading and locally computing. WPT is able to take radio frequency signals as energy sources, and it provides energy for MDs [4]. Therefore, integrating EH and WPT technology into the MEC network system enables systems and MDs to perform long-term stable and sustainable computation, and it also has the potential to improve computing performance [10].

Although quite a lot of researchers have investigated EH, most of these researchers have only considered general EH methods. Comparatively, there is a relative lack of studies that focus on energy-harvesting techniques based on full-duplex and half-duplex modes, and few studies have investigated three-layer heterogeneous MEC network systems simultaneously. Therefore, it is desirable to jointly consider the resource allocation and decision schemes based on a novel full/half-duplex EH modes enabled with heterogeneous MEC network systems.

In this work, we primarily consider a WPT model based on the full/half-duplex transmission technique, which is combined with the designed three-tier heterogeneous MEC architecture. The aim of this work seeks to minimize the overall energy consumption and optimize the offloading decision, EH time allocation and mode selection. We formulate the optimization program as a mixed integer programming problem, which is NP-hard. To address the formulated problem, the main objective can be reformulated as a non-convex quadratically constrained quadratic program (QCQP) form [11], and the separable Semi-Definite Relaxation (SDR) method can be adopted to find the optimal offloading decision strategy and the EH time allocation [12].

Comparing with the recent research work, the main research work and the key contributions of this paper can be summarized as follows:

- In the proposed MEC system, we consider full-duplex and half-duplex WPT modes and analyze the energy consumption problems under these two different energy transfer modes separately with the aim to derive the optimal mode selection strategy.
- In particular, we design a three-tier heterogeneous MEC offloading network architecture that considers the multi-level computational task offloading problem, where the tasks of MDs can be executed locally, offloaded to a server with energy source for computing, or transformed to a remote MEC server for processing;
- In addition, we investigate an optimization problem involving the minimization of transmission and computational energy consumption based on complete time and energy constraints, which is a non-convex mixed-integer programming problem. We derive the optimal offloading decision strategy and WPT mode by adopting QCQP and SDR methods;
- Moreover, we analyze the optimal time of EH for MDs under the half-duplex energy transfer mode with a linear form of WPT. Finally, we propose an iterative algorithm with the aim to solve the joint optimal offloading decision, time allocation of WPT under the half-duplex EH mode, as well as the mode selection.

The reminder of this paper is organized as follows. Section 2 is concerned with giving a brief review of relevant research work. In Section 3, we present the system model. The problem formulation and constraints are proposed in Section 4. In Section 5, we demonstrate the simulation results. Finally, Section 6 concludes this research and discusses future directions.

## 2. Related Work

MEC technology has been widely used in the communication field and different scenarios to provide services to network edge users by taking advantage of mobile edge computing servers closer to users and abundant computing resources [13]. To solve the resource allocation problem in a dynamic computational offload environment, in [14], the authors used a deep reinforcement learning-based approach and proposed a deep deterministic policy gradient (DDPG) dynamic policy to solve the dynamic offload, computational offload and resource allocation problems. With the development of blockchain technology [15], MEC technology is used in blockchain, and MEC technology can be used to solve the challenge of insufficient computational power of MDs in mining tasks [16]. MEC technology has been further applied to vehicle networking for driver assistance services to improve the safety and intelligence of vehicle driving [17]. However, how to ensure that MDs in the MEC network system perform a long time computing task offloading, maintain the MDs with enough power for computing task offloading, and guarantee that the MEC network system can operate for a long time is also one of the main challenges currently faced.

More recently, there has been a growing research interest in exploring the application of EH and WPT in MEC systems [1]. In [18], the authors proposed an actor–critic learning algorithm based on mixed decision making, and a deep reinforcement learning algorithm of multi-MD mixed actor–critic for dynamic computing and offloading was proposed. Similarly, in [19], a deep reinforcement learning-based online offloading algorithm was proposed with the goal of maximizing the weighted sum of computational rates and optimizing the time allocation for the user task offloading decisions scheme and wireless EH. In the single-user MEC system, the authors propose an energy-efficient resource allocation scheme for WPT and task allocation for the local and offloading computation of MDs. Considering the unpredictability of EH and the situation of dynamic offloading of computation tasks, in [6], the authors presented an online algorithm of the Lyapunov method to optimize the stability of the battery queue and minimize the power consumption. Considering the nonlinear energy collection mode [20], the authors investigated the resource allocation to maximize the computational efficiency and optimized the EH time, the CPU cycle frequency of the local computation, the offloading time, and the power allocation to obtain the optimal solution. In [21], in order to minimize the system cost, the authors proposed a distributed optimization problem to solve the optimal offloading, charging and discharging energy and renewable energy utilization rate of MBSs. In [22], the authors investigated the computational offloading and resource allocation problem in IoT networks, which simultaneously support end-device mobility and energy harvesting, and they proposed a Lyapunov optimization and semi-definite programming (SDP)-based online mobility-aware offloading and resource allocation algorithm.

Several studies have revealed that wireless information and power transfer technology provides a potential solution to increase the data transmission rate and long-term service of user equipment in the wireless network. Meanwhile, EH technology can also be implemented to enhance the energy efficiency of network [23,24]. In [23], the authors investigated resource allocation for the orthogonal frequency division multiplexing (OFDM)-based nonorthogonal multiple access (NOMA) system empowered by WPT technology with the aim to obtain secure and energy efficient transmission. Similar to [24], the authors researched an energy-efficient resource allocation for the WPT-enabled orthogonal frequency division multiple access (OFDMA) multicell networks. In order to achieve long battery life, in [25], the authors introduce an online learning algorithm with a distributed execution approach for computational offloading in WP-MEC networks, which aims to minimize the long-term average task completion delay of mobile clients. In [26], the authors combined social networking techniques and EH techniques for enhancing the performance of fog computing systems. In [27], the authors introduced a wireless energy harvesting (WEH)-based industrial Internet of Things (CIIoT) to harvest RF energy from primary user signals, and they proposed energy-efficient resource allocation under different spectrum access methods to maximize the average transmission rate of the CIIoT while maintaining energy-saving requirements. In [28], the authors proposed an MEC offloading scheme for cellular IoT networks with a large number of NOMA-assisted energy harvesting MDs based on social relationships among the user communication devices to optimize the processing mode selection, device clustering, channel and power allocation for the purpose of maximizing the total network rate and under the constraints of power, energy and delay.

With the application and development of wireless networks, it has become an important research field to apply full-duplex and half-duplex technology to the current communication system based on wireless energy transform technology. To solve the resource allocation problem in multi-access edge computation offloading, the full-duplex assisted multi-access edge computation offloading system is investigated in [29], and they proposed a step-wise resource allocation method for enhancing the performance of the computation offloading subject to data rate constraint. In [30], the authors studied an unmanned aerial vehicle (UAV) wireless communication system with EH, where the UAV transmits energy to MDs in half-duplex or full-duplex manner, and the user first collects energy and then offloads the data to the UAV, with the goal of minimizing the total energy consumption of the UAV and achieving minimal delay requirement of the data transmission of the user. Considering the security of wireless communication system, in [31], the authors examined the energy-constraint secrecy performance of a wireless network with passive eavesdroppers and wireless information and power energy transmission, and they proposed a full-duplex automatic jamming scheme. In [32], the authors investigate the problem of opportunity mode selection and user scheduling in both single-carrier and multi-carrier OFDM full-duplex systems with the objective of maximizing the system utility (e.g., sum rate) for long-term and short-term time fairness.

## 3. System Model

**Figure 1:**
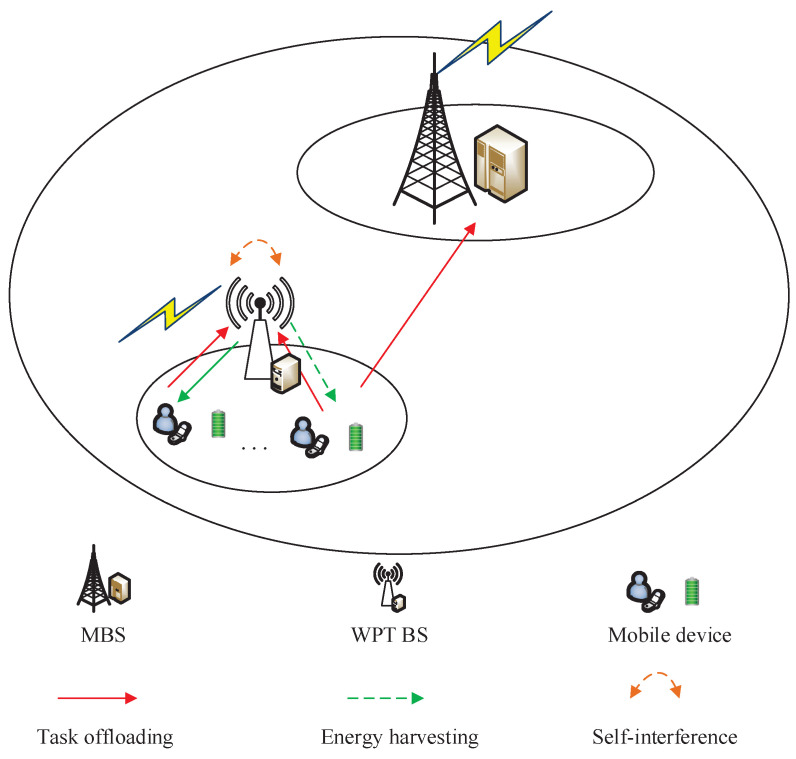
MEC offloading system model for wireless energy transmission.

The three-tier WPT MEC system model is shown in Figure 1. The system model consists of multiple MDs, WPT BS, and MBS. The WPT BS transmits energy to the neighboring users in a broadcast mode, and each MD has a wireless energy-harvesting capability, and the harvested energy is stored. The MD is closer to the WPT BS, while the distance to the MBS is relatively long. Throughout this paper, we use U={1,2,3…,i,…,U}, i∈U to represent the set of MDs. Each MD *i* is equipped with an EH battery, and the computing capacity of *i* is defined as $F_{i}^{l}\in \left[0,F_{i}^{\max}\right]$. Furthermore, MDs have full/half-duplex EH modes, and the harvested energy will be stored in the battery. The WPT BS is represented by WPT BS and adopts full-duplex or half-duplex mode to carry out WPT for MDs, and it can also provide computing services with computing capacity $F^{R}$. In order to enhance the computing capacity of the system, a server with powerful computing capacity is integrated into MBS, and the computing capacity of the MBS server is represented by $F^{M}$. In the three-tier heterogeneous MEC system, MDs are able to choose to perform local computing, either offloading computing tasks to a server at the WPT BS for executing or offloading to a remote MBS server for processing. The uplink between the MD and the WPT BS or MBS employs a wireless connection for the communication and offloading of computation tasks, while in the downlink, the WPT BS and MBS return the processed computation task results to the MD, and since the data size of the computation results is relatively small, hence, we ignore the transmission delay and communication energy consumption of the computation result return in the downlink in this case.

In the proposed MEC system, we suppose that the task of MD *i* is completed within a time interval $t_{i}$. We assume that MDs take the EH and task processing simultaneously based on full-duplex mode. In half-duplex mode, the MD *i* performs EH during time slot $\tau_{i}$ firstly, and then, the task will be processed within time slot $t_{i}-\tau_{i}$. We express the data size of the computing task for MD *i* in terms of $d_{i}$, and *w* represents the number of CPU cycles required to calculate each bit of data. The term $A_{i}=\left[a_{i,l},a_{i,R},a_{i,M}\right],i\in U$ will be used in this paper to refer the set of computing task offloading decision factors. The computational tasks of each MD can be optionally computed locally or be offloaded to the wPT BS or MBS server for computing. Specifically, $a_{i,l}=1$ represents the selection factor calculated locally by the MDs, $a_{i,R}=1$ means that the MD *i* chooses the WPT BS for offloading computation, and $a_{i,M}=1$ indicates that the MDs choose the MBS server for the offloading process; otherwise, $a_{i,l}=a_{i,R}=a_{i,M}=0$. The key notation can be found in Table 1. Due to the fact that the computation task of the MDs can only choose one of the offloading decisions for computing, we can obtain the offloading decision strategy constraints as follows:

(1) $$a_{i,l}+a_{i,R}+a_{i,M}=1,a_{i,l},a_{i,R},a_{i,M}\in \left\{0,1\right\},\forall i\in U.$$

### 3.1. Local Computing Mode

When the local computing is adopted, the processing capacity of MDs can be dynamically adjusted according to the size of the task and the completion time of the task by employing dynamic voltage and frequency expansion technology [18,33]. We assume that the computing capability $F_{i}^{l}$ of the MD *i* remains unchanged. Accordingly, the time for local computation processing can be defined as follows:

(2) $$D_{i}^{t}=\frac{d_{i}w}{F_{i}^{l}}.$$

Furthermore, the energy consumption of local computing of the MD *i* can be expressed as follows:

(3) $$E_{i}^{t}=\omega d_{i}w{\left(F_{i}^{l}\right)}^{2},$$

where ω is the number of effective capacitors related to chip structure [34], and *w* represents the number of CPU cycles required to calculate each bit of data.

### 3.2. WPT BS Server Model

Given that the WPT BS is capable of transmitting energy to the MDs via wireless transmission during the computation offloading, in the current system, we consider the use of half-duplex and full-duplex methods for energy transmission. The distance between MD *i* and WPT BS is $D_{i}^{R}$, and the channel gain between MD *i* and WPT BS is $g_{i,R}={\left(D_{i,r}\right)}^{\sigma }$, where σ=−4 is the path loss factor.

#### 3.2.1. Half-Duplex Mode

In the half-duplex mode [30], WPT BS firstly transmits energy to the MD *i* in the time $\tau_{i}$, where $0<\tau_{i}<t_{i}$ is the time allocation for EH under half-duplex mode. Then, the computational task of MD *i* will be processed locally or offloaded to the server during the remaining time $\left(t_{i}-\tau_{i}\right)$.

With the half-duplex mode, the achievable uplink transmission rate between the MD *i* and the WPT BS can be given by:

(4) $$r_{{}_{i,R}}^{HD}=B_{i,R}\log\left(1+\frac{p_{i,R}g_{i,R}}{I}\right),$$

where $p_{i,R}$ is the transmit power of MD *i* and and $P_{i,R}\leq P_{i}^{\max}$. *I* is the noise power, $B_{i,R}$ is the transmission bandwidth. The harvested energy and transmission energy consumption in half-duplex mode are respectively calculated by [35],

(5) $$e_{i}^{HD}=\varsigma P_{R}\tau_{i},$$

(6) $$E_{i,R}^{HD}=\frac{p_{i,R}d_{i}}{r_{i,R}^{HD}}.$$

#### 3.2.2. Full-Duplex Mode

In full-duplex mode, WPT BS transmits wireless energy to the MD *i*, and the transmit power is $P_{R}$. Meanwhile, the MD *i* can choose to offload the task to the base station for computing within the time through the harvested energy. We consider linear EH in the proposed MEC network system; thus, during time $t_{i}$, the energy harvested by the MD *i* can be given by [35]

(7) $$e_{i}^{FD}=\varsigma P_{R}t_{i}.$$

In full-duplex WPT mode, the WPT BS can simultaneously transmit to and receive task offloading from the MDs. In this case, self-coherent interference is generated. Then, the achievable uplink transmission rate between the MD *i* and the WPT BS becomes:

(8) $$r_{{}_{i,R}}^{FD}=B_{i,R}\log\left(1+\frac{p_{i,R}g_{i,R}}{I+\sigma P_{R}}\right).$$

$P_{R}$ denotes the transmit power of the WPT BS, and σ denotes the effective self-interference coefficient in full-duplex mode [30]. The transmission energy consumption of MD *i* for transmitting to WPT BS can be expressed by

(9) $$E_{i,R}^{FD}=\frac{p_{i,R}d_{i}}{r_{i,R}^{FD}}.$$

### 3.3. MBS Server Model

In the considered system, MDs can also choose to offload computation tasks to an MBS server. The server of MBS is integrated with powerful computing units. Let us assume that the distance between the MD *i* and the MBS is $D_{i,M}$, and the channel gain between the MD *i* and MBS is $g_{i,M}={\left(D_{i,M}\right)}^{\sigma }$. The uplink transmission rate is given as follows:

(10) $$r_{i,M}=B_{i,M}\log\left(1+\frac{p_{i,M}g_{i,M}}{I}\right),$$

where $p_{i,M}$ represents the transmit power of MD *i*, and $B_{i,M}$ is the transmission bandwidth. The transmission energy consumption is given by:

(11) $$E_{i,M}^{w}=\frac{p_{i,M}d_{i}}{r_{i,M}}.$$

The computing energy consumption of the MBS server can be expressed by:

(12) $$E_{i,M}^{c}=\varphi \frac{d_{i}w}{F_{M}},$$

where φ represents the amount of energy consumed per CPU cycle by the MBS server to perform computing tasks for MDs.

## 4. Problem Formulation

In this section, we formulate an optimization problem with the aim to minimize the overall energy consumption of computation tasks of MDs. We optimize the offloading decision strategy $A_{i}$, EH time allocation $\left\{\tau_{i}\right\}$, and full/half-duplex mode optimal decision $x_{i}^{HD},x_{i}^{FD}$ based on the energy constrained of MD *i* and the delay of the computation task. Mathematically, the optimization problem is shown as follows:

(13) $$\begin{array}{cc} \; & P1:\min_{\left\{A_{i},x_{i}^{HD},x_{i}^{FD},\tau_{i}\right\}}\sum_{i=1}^{U}a_{i,l}\omega d_{i}w{\left(F_{i}^{l}\right)}^{2}+a_{i,R}\left(\frac{x_{i}^{HD}p_{i,R}d_{i}}{r_{{}_{i,R}}^{HD}}+\frac{x_{i}^{FD}p_{i,R}d_{i}}{r_{{}_{i,R}}^{FD}}+\frac{\omega d_{i}w}{F_{R}}\right)+a_{i,M}\left(\frac{p_{i,M}d_{i}}{r_{i,M}}+\frac{\varphi d_{i}w}{F_{M}}\right)\\ \; & C1:a_{i,l}+a_{i,R}+a_{i,M}=1,\\ \; & C2:\{a_{i,l},a_{i,R},a_{i,M}\}\in \{01\},\\ \; & C3:x_{i}^{HD}+x_{i}^{FD}=1,\\ \; & C4:\left\{x_{i}^{HD},x_{i}^{FD}\right\}\in \left\{0,1\right\},\\ \; & C5:x_{i}^{FD}\varsigma P_{R}t_{i}+x_{i}^{HD}\varsigma P_{R}\tau_{i}t_{i}-a_{i,l}\omega d_{i}w{\left(F_{i}^{l}\right)}^{2}-a_{i,R}\left(\frac{x_{i}^{HD}p_{i,R}d_{i}}{r_{i,R}^{HD}}+\frac{x_{i}^{FD}p_{i,R}d_{i}}{r_{i,R}^{FD}}\right)-\frac{a_{i,M}p_{i,M}d_{i}}{r_{i,M}}\leq 0.\\ \; & C6:0\leq \tau_{i}\leq t_{i},\\ \; & C7:F_{R}\leq F_{R}^{\max},\\ \; & C8:F_{M}<F_{M}^{\max},\\ \; & C9:F_{i}^{l}\leq F_{i}^{\max},\\ \; & C10:x_{i}^{FD}T_{i}^{F}\leq t_{i},\\ \; & C11:x_{i}^{HD}T_{i}^{H}\leq t_{i}-\tau_{i}, \end{array}$$

where $T_{i}^{F}=a_{i,l}\frac{d_{i}w}{F_{i}^{l}}+a_{i,R}\left(\frac{d_{i}}{r_{i,R}^{FD}}+\frac{d_{i}w}{F_{R}}\right)+a_{i,M}\left(\frac{d_{i}}{r_{i,M}}+\frac{d_{i}w}{F_{M}}\right),$ and $T_{i}^{H}=a_{i,l}\frac{d_{i}w}{F_{i}^{l}}+a_{i,R}\left(\frac{d_{i}}{r_{i,R}^{HD}}+\frac{d_{i}w}{F_{R}}\right)+a_{i,M}\left(\frac{d_{i}}{r_{i,M}}+\frac{d_{i}w}{F_{M}}\right)$.

C1 and C2 represent the constraints of the offloading decision of MD *i*. C3 and C4 indicate the EH mode of MD *i*, $x_{i}^{HD}$ indicates that the MD selects a half-duplex EH mode, $x_{i}^{FD}$ indicates that the MD *i* selects a full-duplex EH mode, and only one of the EH modes decision can be selected. C5 makes sure that the energy consumption is less than the harvested energy. C6 denotes the time allocation for EH with half-duplex mode. C7–C9 are utilized to guarantee that the computing resources allocated to an MD should be limited. C10 indicates that the task processing latency for different task computation and offloading methods under the selected full-duplex mode by the MD *i* is lower than the maximum allowable time slot $t_{i}$. C11 denotes that in half-duplex mode, the MD performs EH first before the task processing in time $\tau_{i}$; i.e., the delay of the task processing cannot exceed the remaining time slot $t_{i}-\tau_{i}$.

The computation offloading decision factor $A_{i}=\left\{a_{i,l},a_{i,R},a_{i,M}\right\}$ and the full-duplex and half-duplex mode decision strategy $x_{i}=\left\{x_{i}^{FD},x_{i}^{HD}\right\}$ are integer variables, and the EH time allocation $\tau_{i}$ is a continuous variable. In this case, we note that the formulated problem P1 is a mixed integer programming problem. The computational complexity of addressing this problem is high. Therefore, we employ an iterative method to solve the optimization problem P1. Firstly, the optimization problem P1 can be transformed into separable QCQP, and then, the method of SDR is used to obtain the binary computing offloading decision and the optimal mode selection [36]. Then, we will use the convex optimization program method to solve the time $\tau_{i}$ for EH based on the half-duplex mode.

### 4.1. Computation Offloading Decision Strategy

We assume that $x_{i}=\left\{x_{i}^{FD},x_{i}^{HD}\right\}$ and $\tau_{i}$ are given. Initially, we can convert the problem P1 with an equivalent QCQP form so as to achieve the SDR formulation finally according to [37].

#### 4.1.1. QCQP Form and SDR

The variable set of $A_{i}$ is integer variable. Thus, we rewrite constraint C2 as follows:

(14) $$C2^{a}:a_{i,l}\left(a_{i,l}-1\right)=0,a_{i,R}\left(a_{i,R}-1\right)=0,a_{i,M}\left(a_{i,M}-1\right)=0,$$

P1 can be formulated as follows:

$$\begin{array}{ccc} P2: & \min_{A_{i}}\sum_{i=1}^{U}a_{i,l}\omega d_{i}w{\left(F_{i}^{l}\right)}^{2}+a_{i,R}(\frac{x_{i}^{HD}p_{i,R}d_{i}}{r_{{}_{i,R}}^{HD}}+\; & \frac{x_{i}^{FD}p_{i,R}d_{i}}{r_{{}_{i,R}}^{FD}}+\frac{\omega d_{i}w}{F_{R}})+a_{i,M}\left(\frac{p_{i,M}d_{i}}{r_{i,M}}+\frac{\varphi d_{i}w}{F_{M}}\right) \end{array}$$

(15) $$\begin{array}{cc} \; & C1:a_{i,l}+a_{i,R}+a_{i,M}=1,\\ \; & C2^{a}:a_{i,l}\left(a_{i,l}-1\right)=0,a_{i,R}\left(a_{i,R}-1\right)=0,a_{i,M}\left(a_{i,M}-1\right)=0,\\ \; & C5:a_{i,l}\omega d_{i}w{\left(F_{i}^{l}\right)}^{2}+a_{i,R}\left(\frac{x_{i}^{HD}p_{i,R}d_{i}}{r_{i,R}^{HD}}+\frac{x_{i}^{FD}p_{i,R}d_{i}}{r_{i,R}^{FD}}\right)+\frac{a_{i,M}p_{i,M}d_{i}}{r_{i,M}}\leq x_{i}^{FD}\varsigma P_{R}t_{i}+x_{i}^{HD}\varsigma P_{R}\tau_{i}t_{i},\\ \; & C10,C11. \end{array}$$

The problem P2 is still non-convex, and it is difficult to be solved as $C2^{a}$ are non-convex quadratic constraints. In the following, the problem is transformed into a convex problem based on QCQP transformation, and then, we adopt SDR to obtain the fractional solution.

We vectorized the parameters and variables in P2 as

(16) $$v={\left[a_{1,l},a_{1,R},a_{1,M},\ldots ,a_{U,l},a_{U,R},a_{U,M}\right]}^{T},$$

(17) $$\begin{array}{c} u_{i}^{\prime }=[\omega d_{1}w{\left(F_{i}^{l}\right)}^{2},\frac{x_{1}^{HD}p_{1,R}d_{i}}{r_{{}_{1,R}}^{HD}}+\frac{x_{1}^{FD}p_{1,R}d_{1}}{r_{{}_{1,R}}^{FD}}+\frac{\omega d_{1}w}{F_{R}},\frac{p_{1,M}d_{1}}{r_{1,M}}+\frac{\varphi d_{1}w}{F_{M}},\ldots ,\\ \;\omega d_{U}w{\left(F_{i}^{l}\right)}^{2},\frac{x_{U}^{HD}p_{U,R}d_{U}}{r_{{}_{1,R}}^{HD}}+\frac{x_{U}^{FD}p_{U,R}d_{U}}{r_{{}_{U,R}}^{FD}}+\frac{\omega d_{U}w}{F_{R}},\frac{p_{U,M}d_{U}}{r_{U,M}}+\frac{\varphi d_{U}w}{F_{M}}]. \end{array}$$

Accordingly, the problem P2 is converted into an equivalent QCQP problem as follows:

$$P3:\min_{v}\sum_{i=1}^{U}{\left(u_{i}^{\prime }\right)}^{T}v$$

(18) $$\begin{array}{cc} \; & C1^{\prime }:{\left(u_{i}\right)}^{T}v=1,\;\;\forall i\in U,\\ \; & C2^{\prime }:v^{T}diag\left(e_{j}\right)v-{\left(e_{j}\right)}^{T}v=0,\;j=1,\ldots ,3U,\\ \; & C5^{\prime }:{\left(u_{i}^{e}\right)}^{T}v⩽\left(x_{i}^{FD}\varsigma P_{R}t_{i}\;+\;x_{i}^{HD}\varsigma P_{R}\tau_{i}t_{i}\right),\;\;\forall i\in U,\\ \; & C10^{\prime }:{\left(u_{i}^{F}\right)}^{T}v⩽t_{i},\;\;\forall i\in U,\\ \; & C11^{\prime }:{\left(u_{i}^{H}\right)}^{T}v⩽t_{i}-\tau_{i},\;\;\forall i\in U. \end{array}$$

where

(19) $$\begin{array}{cc} \; & u_{i}=e_{3i-2}+e_{3i-1}+e_{3i}, \end{array}$$

(20) $$\begin{array}{cc} \; & u_{i}^{e}\;=\;[\omega d_{i}w{\left(F_{i}^{l}\right)}^{2},\frac{x_{i}^{HD}p_{i,R}d_{i}}{r_{i,R}^{HD}}+\frac{x_{i}^{FD}p_{i,R}d_{i}}{r_{i,R}^{FD}},\frac{a_{i,M}p_{i,M}d_{i}}{r_{i,M}}], \end{array}$$

(21) $$\begin{array}{cc} \; & u_{i}^{F}\;=\;x_{i}^{FD}[\frac{d_{i}w}{F_{i}^{l}},\frac{d_{i}}{r_{i,R}^{FD}}\;+\;\frac{d_{i}w}{F_{R}},\frac{d_{i}}{r_{i,M}}\;+\;\frac{d_{i}w}{F_{M}}], \end{array}$$

(22) $$\begin{array}{cc} \; & u_{i}^{H}=x_{i}^{HD}\left[\frac{d_{i}w}{F_{i}^{l}},\frac{d_{i}}{r_{i,R}^{HD}}\;+\;\frac{d_{i}w}{F_{R}},\frac{d_{i}}{r_{i,M}}\;+\;\frac{d_{i}w}{F_{M}}\right]. \end{array}$$

However, the formulated problem of P3 is still a non-convex separable QCQP problem which results in the problem remaining intractable. For the purpose of finding approximate solutions, we utilize SDR, which is an effective method to simplify the QCQP problem [36].

#### 4.1.2. Semi-Definite Relaxtion

We define a semi-definite matrix $V\;=\;[v^{T},1{]}^{T}\left[v^{T},1\right]$ of rank one. The problem P3 will be relaxed into a separable semi-definite programming problem as shown below:

$$P3^{\prime }:\;\min_{V}\sum_{i=1}^{U}\mathrm{Tr}(\mathrm{ZV})$$

(23) $$\begin{array}{cc} \; & C1^{\prime \prime }:\mathrm{Tr}\left(Z_{i}V\right)=1,\forall i\in U,\\ \; & C2^{\prime \prime }:\mathrm{Tr}\left(Z_{j}V\right)=1,j=1,2,\ldots ,3U,\\ \; & C5^{\prime \prime }:\mathrm{Tr}\left(Z_{i}^{e}V\right)⩽\left(x_{i}^{FD}\varsigma P_{R}t_{i}\;+\;x_{i}^{HD}\varsigma P_{R}\tau_{i}t_{i}\right),\\ \; & C10^{\prime \prime }:\mathrm{Tr}\left(Z_{i}^{F}V\right)⩽t_{i},\\ \; & C11^{\prime \prime }:\mathrm{Tr}\left(Z_{i}^{H}V\right)⩽t_{i}-\tau_{i},\\ \; & C12:V(3U+1,3U+1)=1,\\ \; & C13:V⪰0,\\ \; & C14:\mathrm{rank}(V)=1. \end{array}$$

where

$$\begin{array}{cccc} Z_{i} & =\left[\begin{array}{cc} 0_{3U} & \frac{1}{2}u_{i}\\ \frac{1}{2}{\left(u_{i}\right)}^{T} & 0_{3U} \end{array}\right], & Z_{i}^{e}= & \left[\begin{array}{cc} 0_{3U} & \frac{1}{2}u_{i}^{e}\\ \frac{1}{2}{\left(u_{i}^{e}\right)}^{T} & 0_{3U} \end{array}\right],\\ Z_{j} & =\left[\begin{array}{cc} \mathrm{diag}(e_{j}) & -\frac{1}{2}e_{j}\\ \frac{1}{2}{\left(e_{j}\right)}^{T} & 0 \end{array}\right], & Z= & \left[\begin{array}{cc} 0_{3U} & \frac{1}{2}{u^{\prime }}_{i}\\ \frac{1}{2}{\left({u^{\prime }}_{i}\right)}^{T} & 0_{3U} \end{array}\right],\\ Z_{i}^{F} & =\left[\begin{array}{cc} 0_{3U} & \frac{1}{2}u_{i}^{F}\\ \frac{1}{2}{\left(u_{i}^{F}\right)}^{T} & 0_{3U} \end{array}\right], & Z_{i}^{H}= & \left[\begin{array}{cc} 0_{3U} & \frac{1}{2}u_{i}^{H}\\ \frac{1}{2}{\left(u_{i}^{H}\right)}^{T} & 0_{3U} \end{array}\right]. \end{array}$$

$P3^{\prime }$ is still non-convex due to the rank constraint C14. The problem can be relaxed to a semi-positive definite programming problem by discarding the rank constraint as follows:

$$\begin{array}{c} P3^{\prime \prime }:\;\min_{V}\sum_{i=1}^{U}\mathrm{Tr}(\mathrm{ZV}) \end{array}$$

(24) $$s.t.\;\;\;C1^{\prime \prime },C2^{\prime \prime },C5^{\prime \prime },C10^{\prime \prime },C11^{\prime \prime },C12,C13.$$

Therefore, the original problem is reformulated to the standard convex optimization problem, and we adopt standard CVX tools to address problem $P3^{\prime \prime }$ [38].

#### 4.1.3. Extracting Offloading Decision

Hereafter, we extract a feasible solution v for P3 from the global optimal solution $V^{*}$ of $P3^{\prime \prime }$, and we obtain the optimal offloading decision from the feasible solution v according to [39,40].

According to the definition of V, we know that only the submatrix of $V^{*}$ topmost coordinates 3N×3N, defined as $V^{\prime }*$, is necessary to obtain the offloading decision $A_{i}$, and that all diagonal elements of $V^{*}$ are positive numbers from 0 to 1. Let us define $pr=[pr_{1,l},pr_{1,R},pr_{1,M},\ldots ,pr_{U,l},pr_{U,R},pr_{U,M}{]}^{T}\overset{\Delta }{\;=}diag\left({V^{\prime }}^{*}\right)$, where each of these terms pr represents the probability of corresponding entry of $A_{i}$ being 1.

We define $K_{i,l}=pr_{i,l}\left(1-pr_{i,R}\right)\left(1-pr_{i,M}\right)$, $K_{i,R}=pr_{i,R}\left(1-pr_{i,l}\right)\left(1-pr_{i,M}\right)$, $K_{i,M}=pr_{i,M}\left(1-pr_{i,l}\right)\left(1-pr_{i,R}\right)$ to satisfy the constraint $a_{i,l}+a_{i,R}+a_{i,M}=1$. According to the probabilities of local computing, the WPT BS server execution and the MEC server execution are expressed as $Pr_{i,l}=K_{i,l}/\left(K_{i,l}+K_{i,R}+K_{i,M}\right)$, $Pr_{i,R}=K_{i,R}/\left(K_{i,l}+K_{i,R}+K_{i,M}\right)$, $Pr_{i,M}=K_{i,M}/\left(K_{i,l}+K_{i,R}+K_{i,M}\right)$, respectively. The computation task offloading decision of MD *i* is given in the following:

(25) $$O_{n}=\left\{\begin{array}{c} \left(1,0,0\right)\\ \left(0,1,0\right)\\ \left(0,0,1\right) \end{array}\right\}.$$

$O_{n}=\left(1,0,0\right)$ means that the computation task will be calculated with probability $Pr_{i,l}$ in local computing.

$O_{n}=\left(0,1,0\right)$ shows that the computation task can be calculated with probability $Pr_{i,R}$ in the WPT BS server.

$O_{n}=\left(0,0,1\right)$ defines the computation task will be computed with probability $Pr_{i,M}$ in the MBS server.

As a result, by randomly setting the value of the vector according to (25), P3 can be resolved and the offloading decisions $a_{i,l},a_{i,R},a_{i,M}$ can be obtained.

### 4.2. Time Allocation on the Half-Duplex Mode

In this part, we will dedicate to solve the EH time allocation in half-duplex mode based on a given offloading decision $A_{i}$ and EH pattern $x_{i}$. Based on half-duplex mode, $x_{i}^{HD}=1$. The MD performs EH and task calculation independently. Considering that in the half-duplex mode, the time of $\tau_{i}$ is used for EH, and that the remaining time of $\left(t_{i}-\tau_{i}\right)$ is used for task offloading and computing, the energy consumption includes the energy consumption of local computation and the energy consumption of computation task processing. The energy consumption problem is shown below:

$$\begin{array}{cc} P4:\min_{\left\{\tau_{i}\right\}}\sum_{i=1}^{U} & a_{i,l}\omega d_{i}w{\left(F_{i}^{l}\right)}^{2}+a_{i,R}\left(\frac{p_{i,R}d_{i}}{r_{{}_{i,R}}^{HD}}+\frac{\omega d_{i}w}{F_{R}}\right)+a_{i,M}\left(\frac{p_{i,M}d_{i}}{r_{i,M}}+\frac{\varphi d_{i}w}{F_{M}}\right) \end{array}$$

(26) $$\begin{array}{cc} \; & C5:\varsigma P_{R}\tau_{i}-a_{i,l}\omega d_{i}w{\left(F_{i}^{l}\right)}^{2}-\frac{a_{i,R}p_{i,R}d_{i}}{r_{i,R}^{HD}}-\frac{a_{i,M}p_{i,M}d_{i}}{r_{i,M}}\geq 0,\\ \; & C6:0<\tau_{i}\leq t_{i},\\ \; & C11:T_{i}^{H}\leq t_{i}-\tau_{i}, \end{array}$$

where

(27) $$T_{i}^{H}=a_{i,l}\frac{d_{i}w}{F_{i}^{l}}+a_{i,R}\left(\frac{d_{i}}{r_{i,R}^{HD}}+\frac{d_{i}w}{F_{R}}\right)+a_{i,M}\left(\frac{d_{i}}{r_{i,M}}+\frac{d_{i}w}{F_{M}}\right).$$

Due to the fact that the objective function is independent of the time variable $\tau_{i}$, we represent the energy consumption in the objective function of P4 with $\psi_{i}^{HD}$, thus

$$\begin{array}{cc} \psi_{i}^{HD}= & a_{i,l}\omega d_{i}w{\left(F_{i}^{l}\right)}^{2}+a_{i,R}\frac{\omega d_{i}w}{F_{R}}+a_{i,M}\left(\frac{p_{i,M}d_{i}}{r_{i,M}}+\frac{\varphi d_{i}w}{F_{M}}\right)+\frac{a_{i,R}p_{i,R}d_{i}}{r_{{}_{i,R}}^{HD}}. \end{array}$$

The constraint C3 can be expressed as:

(28) $$C5^{h}:\varsigma P_{R}\tau_{i}+a_{i,R}\frac{\omega d_{i}w}{F_{R}}+a_{i,M}\frac{\varphi d_{i}w}{F_{M}}-\psi_{i}^{HD}\geq 0.$$

Problem P4 can be rewritten to the problem $P4^{\prime }$ as follows:

$$P4^{\prime }:\min_{\left\{\tau_{i},\psi_{i}^{HD}\right\}}\sum_{i}^{U}\psi_{i}^{HD}$$

(29) $$s.t.\;\;\;C5^{h},C6,C11.$$

To resolve the problem P4’, so that $\psi_{i}^{HD}$ achieves the minimum value and the transmission and computation time constraints of the computation task are met, we use tight operation, let

(30) $$\varsigma P_{R}\tau_{i}+a_{i,R}\frac{\omega d_{i}w}{F_{R}}+a_{i,M}\frac{\varphi d_{i}w}{F_{M}}=\psi_{i}^{HD}.$$

Then, problem $P4^{\prime }$ can be transformed into the following form of problem $P4^{\prime \prime }$:

$$P4^{\prime \prime }:\min_{\left\{\tau_{i}\right\}}\;\;\sum_{i=1}^{U}\varsigma P_{R}\tau_{i}+a_{i,R}\frac{\omega d_{i}w}{F_{R}}+a_{i,M}\frac{\varphi d_{i}w}{F_{M}}$$

(31) s.t.C5,C6,C11.

From the constraints C3 and C9, the dominant of $\tau_{i}$ can be expressed as:

(32) $$\tau_{i}\leq t_{i}-a_{i,l}\frac{d_{i}w}{F_{i}^{l}}-a_{i,R}\left(\frac{d_{i}}{r_{i,R}^{HD}}\;+\;\frac{d_{i}w}{F_{R}}\right)-a_{i,M}\left(\frac{d_{i}}{r_{i,M}}\;+\;\frac{d_{i}w}{F_{M}}\right),$$

(33) $$\tau_{i}\geq \left(a_{i,l}\omega d_{i}w{\left(F_{i}^{l}\right)}^{2}+\frac{a_{i,R}p_{i,R}d_{i}}{r_{i,R}^{HD}}+\frac{a_{i,M}p_{i,M}d_{i}}{r_{i,M}}\right)/\varsigma P_{R}.$$

The objective function and constraint conditions in $P4^{\prime \prime }$ are linear about the variables $\tau_{i}$, so it is a convex optimization problem. The minimum optimal value is obtained at the boundary point, and the optimal value of $\tau_{i}$ is:

(34) $$\tau_{i}^{*}=\min\left\{a_{i},b_{i}\right\},$$

(35) $$a_{i}\;=\;t_{i}-a_{i,l}\frac{d_{i}w}{F_{i}^{l}}-a_{i,R}\left(\frac{d_{i}}{r_{i,R}^{HD}}\;+\;\frac{d_{i}w}{F_{R}}\right)-a_{i,M}\left(\frac{d_{i}}{r_{i,M}}\;+\;\frac{d_{i}w}{F_{M}}\right),$$

(36) $$b_{i}\;=\;\left(a_{i,l}\omega d_{i}w{\left(F_{i}^{l}\right)}^{2}+\frac{a_{i,R}p_{i,R}d_{i}}{r_{i,R}^{HD}}+\frac{a_{i,M}p_{i,M}d_{i}}{r_{i,M}}\right)/\varsigma P_{R}.$$

### 4.3. Full/Half-Duplex Mode Selection

For the given solution of $A_{i}$ and $\tau_{i}$, we can solve the selection decision of full/half-duplex EH model. Similar to the optimize offloading decision strategy, the variables of the full/half-duplex EH mode decision are still integer variables. Thus, we determine the main objective problem, which is related to variables about $x_{i}=\left\{x_{i}^{FD},x_{i}^{HD}\right\}$; then, the objective problem will be converted to the QCQP form, and we adopt the SDR to obtain the fractional solution.

$$\begin{array}{c} P5:\min_{\left\{x_{i}^{HD},x_{i}^{FD}\right\}}\sum_{i=1}^{U}a_{i,l}\omega d_{i}w{\left(F_{i}^{l}\right)}^{2}+a_{i,R}\left(\frac{x_{i}^{HD}p_{i,R}d_{i}}{r_{{}_{i,R}}^{HD}}+\frac{x_{i}^{FD}p_{i,R}d_{i}}{r_{{}_{i,R}}^{FD}}+\frac{\omega d_{i}w}{F_{R}}\right)+a_{i,M}\left(\frac{p_{i,M}d_{i}}{r_{i,M}}+\frac{\varphi d_{i}w}{F_{M}}\right) \end{array}$$

(37) $$\begin{array}{cc} \; & C3:x_{i}^{HD}+x_{i}^{FD}=1,\\ \; & C4:\;\left\{x_{i}^{HD},x_{i}^{FD}\right\}\in \left\{0,1\right\},\\ \; & C5:x_{i}^{FD}(\varsigma P_{R}t_{i}-\frac{a_{i,R}p_{i,R}d_{i}}{r_{i,R}^{FD}})\;+\;x_{i}^{HD}\left(\varsigma P_{R}\tau_{i}t_{i}-\frac{a_{i,R}p_{i,R}d_{i}}{r_{i,R}^{HD}}\right)⩽a_{i,l}\omega d_{i}w{\left(F_{i}^{l}\right)}^{2}+\frac{a_{i,M}p_{i,M}d_{i}}{r_{i,M}},\\ \; & C10:x_{i}^{FD}T_{i}^{F}\leq t_{i},\\ \; & C11:x_{i}^{HD}T_{i}^{H}\leq t_{i}-\tau_{i}. \end{array}$$

We can reformulate the constraint of C4 as follows:

(38) $$\begin{array}{cc} C4^{\left(b\right)}: & x_{i}^{HD}\left(x_{i}^{HD}-1\right)=0,x_{i}^{FD}\left(x_{i}^{FD}-1\right)=0. \end{array}$$

The problem P5 can be formulated as shown:

$$\begin{array}{cc} P6: & \min_{\left\{x_{i}^{HD},x_{i}^{FD}\right\}}\sum_{i=1}^{U}a_{i,l}\omega d_{i}w{\left(F_{i}^{l}\right)}^{2}+a_{i,R}\left(\frac{x_{i}^{HD}p_{i,R}d_{i}}{r_{{}_{i,R}}^{HD}}+\frac{x_{i}^{FD}p_{i,R}d_{i}}{r_{{}_{i,R}}^{FD}}+\frac{\omega d_{i}w}{F_{R}}\right)+a_{i,M}\left(\frac{p_{i,M}d_{i}}{r_{i,M}}+\frac{\varphi d_{i}w}{F_{M}}\right) \end{array}$$

(39) $$s.t.\;C3,C4^{\left(b\right)},C5,C10,C11.$$

Due to the fact that the constraint $C4^{\left(b\right)}$ is a non-convex quadratic constraint, the problem P6 is still non-convex and challenging to solve. In order to obtain the optimum decision strategy, we transform the problem into QCQP form initially and SDR to obtain the fractional solution.

#### 4.3.1. QCQP Form

By defining $x=[x_{1}^{HD},x_{1}^{FD},\ldots ,x_{U}^{HD},x_{U}^{FD}]$, the problem can be converted into an equivalent QCQP problem as below:

$$P7:\min_{x}\sum_{i=1}^{U}{\left(q_{i}^{\prime }\right)}^{T}x\;+\;\sum_{i=1}^{U}E_{i}$$

(40) $$\begin{array}{cc} \; & C3^{\prime }:{\left(q_{i}\right)}^{T}x\;=\;1,\forall i\in U,\\ \; & C4^{\prime }:x^{T}diag\left(e_{k}\right)x-{\left(e_{k}\right)}^{T}x=0,\;k=1,\ldots ,2U,\\ \; & C5^{\prime }:{\left(q_{i}^{a}\right)}^{T}x\leq a_{i,l}\omega d_{i}w{\left(F_{i}^{l}\right)}^{2}+\frac{a_{i,M}p_{i,M}d_{i}}{r_{i,M}},\\ \; & C10^{\prime }:{\left(q_{i}^{F}\right)}^{T}x\leq t_{i},\\ \; & C11^{\prime }:{\left(q_{i}^{H}\right)}^{T}x\leq t_{i}-\tau_{i}. \end{array}$$

where

(41) $$E_{i}=a_{i,M}\left(\frac{p_{i,M}d_{i}}{r_{i,M}}+\frac{\varphi d_{i}w}{F_{M}}\right)+\frac{\omega d_{i}w}{F_{R}}+a_{i,l}\omega d_{i}w{\left(F_{i}^{l}\right)}^{2},$$

(42) $$\begin{array}{c} q_{i}^{\prime }={\left[\frac{a_{1,R}p_{1,R}d_{1}}{r_{{}_{1,R}}^{HD}},\frac{a_{1,R}p_{1,R}d_{1}}{r_{{}_{1,R}}^{FD}},\ldots ,\frac{a_{U,R}p_{U,R}d_{U}}{r_{{}_{U,R}}^{HD}},\frac{a_{U,R}p_{U,R}d_{U}}{r_{{}_{U,R}}^{FD}}\right]}^{T}, \end{array}$$

(43) $$\begin{array}{cc} \; & q_{i}\;=\;e_{2i-1}+e_{2i},q_{i}^{F}\;=\;[T_{i}^{F},0{]}^{T},q_{i}^{H}\;=\;[T_{i}^{H},0{]}^{T}, \end{array}$$

(44) $$\begin{array}{cc} \; & q_{i}^{a}\;=\;[\varsigma P_{R}t_{i}-\frac{a_{i,R}p_{i,R}d_{i}}{r_{i,R}^{FD}},\varsigma P_{R}\tau_{i}-\frac{a_{i,R}p_{i,R}d_{i}}{r_{i,R}^{HD}}]. \end{array}$$

Similarly, the QCQP formulation is still a non-convex separable QCQP problem and hard to be solved. Therefore, we take the SDR method to simply the QCQP problem.

#### 4.3.2. Semi-Definite Relaxtion

In order to utilize the SDR method, we define $X\;=\;[x^{T},1{]}^{T}[x^{T},1]$, X is a rank one symmetric positive semi-definite matrix and disregard the constant term $\sum_{i=1}^{U}E_{i}$ from the objective function of P7. Hence, we can obtain the equivalent form as follows:

$$P7^{\prime }:\min_{X}\mathrm{Tr}\left(\mathrm{QX}\right)$$

(45) $$\begin{array}{cc} \; & C3^{\prime \prime }:\mathrm{Tr}(Q_{i}X)⩽a_{i,l}\omega d_{i}w{\left(F_{i}^{l}\right)}^{2}+\frac{a_{i,M}p_{i,M}d_{i}}{r_{i,M}},\\ \; & C4^{\prime \prime }:\mathrm{Tr}\left(Q_{k}X\right)=0,\\ \; & C5^{\prime \prime }:\mathrm{Tr}\left(Q_{i}^{a}X\right)=0,\\ \; & C10^{\prime \prime }:\mathrm{Tr}\left(Q_{i}^{F}X\right)=0,\\ \; & C11^{\prime \prime }:\mathrm{Tr}\left(Q_{i}^{H}X\right)=0,\\ \; & C15:X(2U,2U)=1,\\ \; & C16:X⪰0,\\ \; & C17:\mathrm{rank}(V)=1. \end{array}$$

where

$$\begin{array}{cc} \; & Q_{i}=\left[\begin{array}{cc} 0_{2U\times 2U} & \frac{1}{2}q_{i}\\ \frac{1}{2}{\left(q_{i}\right)}^{T} & 0_{2U\times 2U} \end{array}\right],Q_{k}=\left[\begin{array}{cc} \mathrm{diag}(e_{k}) & -\frac{1}{2}e_{k}\\ \frac{1}{2}{\left(e_{k}\right)}^{T} & 0 \end{array}\right],\\ \; & Q_{i}^{a}=\left[\begin{array}{cc} 0_{2U\times 2U} & \frac{1}{2}q_{i}^{a}\\ \frac{1}{2}{\left(q_{i}^{a}\right)}^{T} & 0_{2U\times 2U} \end{array}\right],Q=\left[\begin{array}{cc} 0_{2U\times 2U} & \frac{1}{2}{q^{\prime }}_{i}\\ \frac{1}{2}{\left({q^{\prime }}_{i}\right)}^{T} & 0_{2U\times 2U} \end{array}\right], \end{array}$$

$$Q_{i}^{F}=\left[\begin{array}{cc} 0_{2U\times 2U} & \frac{1}{2}q_{i}^{F}\\ \frac{1}{2}{\left(q_{i}^{F}\right)}^{T} & 0_{2U\times 2U} \end{array}\right],Q_{i}^{H}=\left[\begin{array}{cc} 0_{2U\times 2U} & \frac{1}{2}q_{i}^{H}\\ \frac{1}{2}{\left(q_{i}^{H}\right)}^{T} & 0_{2U\times 2U} \end{array}\right].$$

However, in problem $P7^{\prime }$, the rank constraint (45) is the only non-constraint. Therefore, we will relax problem $P7^{\prime }$ into a semi-definite programming problem by dropping the rank constraint as follows:

$$P7^{\prime \prime }:\min_{X}\mathrm{Tr}\left(\mathrm{QX}\right)$$

(46) $$\begin{array}{c} s.t.\;C3^{\prime \prime },C4^{\prime \prime },C5^{\prime \prime },C10^{\prime \prime },C11^{\prime \prime },C15,C16. \end{array}$$

Now, we have reformulated the problem into a standard convex optimization problem, and it can be solved in polynominal time with standard CVX tools such as SeDuMi [36].

#### 4.3.3. Full/Half-Duplex Mode Decision Extraction

In this part, we extract a feasible solution x from the global optimal solution $X^{*}$ of the above problem, and we obtain the optimal offloading decision in feasible solution x according to the method proposed in [39,40].

According to the definition of X, we know that only the submatrix of $X^{*}$ whose topmost coordinate is 2N×2N, defined as $X^{*}$, is necessary to obtain the optimal full/half-duplex decision x, and that all diagonal elements of $X^{\prime *}$ are positive numbers from 0 to 1. We define $p={\left[p_{1,f},p_{1,h},\ldots ,p_{U,f},p_{U,h}\right]}^{T}\overset{\Delta }{\;=}\mathrm{diag}\left(X^{\prime *}\right)$, where each term of *p* represents the probability of corresponding term of $x_{i}$ being 1.

In order to satisfy $x_{i}^{HD}+x_{i}^{FD}=1$, we define $K_{i,f}=p_{i,f}\left(1-p_{i,h}\right)$, and based on this, the probabilities of full/half-duplex are $p_{i,f}=K_{i,f}/\left(K_{i,f}+K_{i,h}\right)$ and $p_{i,h}=K_{i,h}/\left(K_{i,f}+K_{i,h}\right)$, respectively.

According to the above solution, the full/half-duplex EH mode decision of MD *i* is given by the following formula:

(47) $$\Omega_{i}=\left\{\begin{array}{c} \left(1,0\right)\\ \left(0,1\right) \end{array}\right\}.$$

$\Omega_{i}=\left(1,0\right)$ means that we choose half-duplex mode with probability $p_{i,f}$;

$\Omega_{i}=\left(0,1\right)$ means that we choose full-duplex mode with probability $p_{i,h}$.

By randomly setting the value of the vector according to the probabilities in (47), P6 can be resolved, and we can obtain the offloading decisions $x_{i}^{HD},x_{i}^{FD}$.

### 4.4. Algorithm Analysis

To this end, we design an algorithm for optimizing the offloading decision strategy, EH time allocation and full/half-duplex mode optimization based on the proposed approach. First of all, the system parameters of computation tasks and network are initialized. Since the offloading decision is randomly obtained based on the obtained probability, we can run the aforementioned procedure several times to obtain a more precise decision. Based on the given EH mode $x_{i}$ and EH time allocation $\tau_{i}$, we can obtain $A_{i}^{n}$, which is denoted as the offloading decision for the *n*th time. Then, based on the $A_{i}^{n}$ and $\tau_{i}$, we can perform EH mode optimization. After that, based on $A_{i}^{n}$ and ${x_{i}}^{n}$, we can carry out half-duplex EH time allocation, and the solution with smallest energy consumption target value is the final solution. The complexity of the internal for-loop is O(N), and the external iterative loops are iterations needed are $O(N^{6})$ and $O(N^{4})$, so the overall complexity is calculated as $O\left(N^{7}\right)=O\left(\left(N^{6}+N^{4}\right)N\right)$. We present the overall algorithm in Algorithm 1.
**Algorithm 1** Jointly optimize the computation offloading decision, EH time allocation and mode selection 1:**Initialize:** 2:Initialize $t_{i}$, $F_{i}^{l}$, $F^{R}$, $F^{M}$, $d_{i}$, $p_{i,R}$, $p_{i,M}$, $P_{R}$. 3:Initialize all the matrixes involved in $P3^{\prime \prime }$ and $P7^{\prime \prime }$; 4:**Iteration:** 5:Solve the SDR problem $P3^{\prime \prime }$ by using the standard CVX tool SeDuMi and to obtain optimal solution of $V^{\prime }*$. 6:Extract the top left corner 3N×3N sub-matrix $V^{\prime }*$ from matrix $V^{*}$ and define the values of diagonal elements in $V^{\prime }*$ as $pr=[pr_{1,l},pr_{1,R},pr_{1,M},\ldots ,pr_{U,l},pr_{U,R},pr_{U,M}{]}^{T}$. 7:Solve the SDR problem $P7^{\prime \prime }$ by using the standard CVX tool SeDuMi and to obtain optimal solution of $X^{\prime *}$. 8:Extract the top left corner 2N×2N sub-matrix $X^{\prime *}$ from matrix $X^{*}$, and define the values of diagonal elements in $X^{\prime *}$ as $p={\left[p_{1,f},p_{1,f},\ldots ,p_{U,f},p_{U,h}\right]}^{T}$. 9:**for** l=1,2,…,L **do**10:    Extract ${A_{i}}^{n}$ from $pr\left(n\right)=[pr_{1,l},pr_{1,R},pr_{1,M},\ldots ,pr_{N,l},$$pr_{U,R},pr_{U,M}{]}^{T}\left(n\right)$ according to (25).11:    Extract ${x_{i}}^{n}$ from $p\left(n\right)={\left[p_{1,f},p_{1,h},\ldots ,p_{U,f},p_{U,h}\right]}^{T}\left(n\right)$ according to (47).12:    Perform EH time allocation $\left\{\tau_{i}\left(n\right)\right\}$ based on half-duplex mode, respectively.13:    Compare the objective value of all the *N* solutions, and select the solution with minimum objective value.14:**end for**15:**Output:** The optimal solutions of the offloading strategy $A_{i}^{*}$, EH time allocation $\tau_{i}^{*}$ and the EH mode scheme $x_{i}^{*}$.

## 5. Numerical Results

In this section, we conduct extensive simulations to verify the feasibility and superiority of the proposed scheme. In this simulation, we used a hardware setup with a desktop computer with 8 G memory capacity, hard disk data size of 1 T, 3.20 GHz CPU frequency, and 64-bit Windows system as the operating system. The software used was MATLAB software for the simulation experiments. We set the number of MDs to 10, one WPT BS, and a macro base station with an integrated powerful server. We set the data size for any task between 10 and 110 KB, and the maximum transmission power of MDs was 30 dbm. The bandwidth size between the MDs and the WPT BS was set to be 20 MHZ, and the bandwidth between the MDs and the MBS was 50 MHZ. The average distance between the MDs and the WPT BS was 100 m, and the maximum distance between the MDs and the MBS is 500 m. We set the transmission power of the WPT BS as 40 dbm.

**Figure 2:**
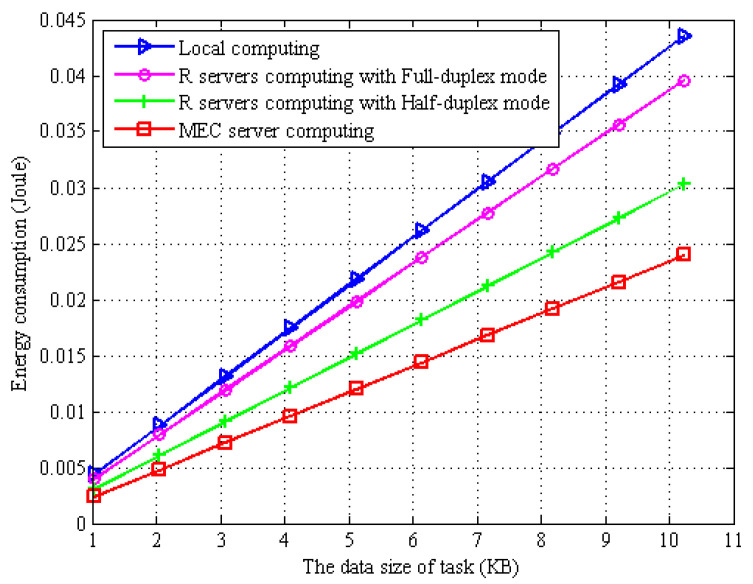
The energy consumption vs. different task size and different task computing.

In Figure 2, we plot the energy consumption of the computation tasks with different data sizes when adopting different computing strategies. From Figure 2, we can see that the energy consumption of all different computation methods increases as the size of the task keeps increasing. Under the local computing approach, the MD is limited by the battery capacity and computing capability, which consumes more energy compared to offloading the task to WPT BS and the MBS server for computing. Self-interference will be generated when MDs employ full-duplex EH technology, which affects the transmission rate of task offloading, thus increasing the transmission energy consumption during task offloading.

**Figure 3:**
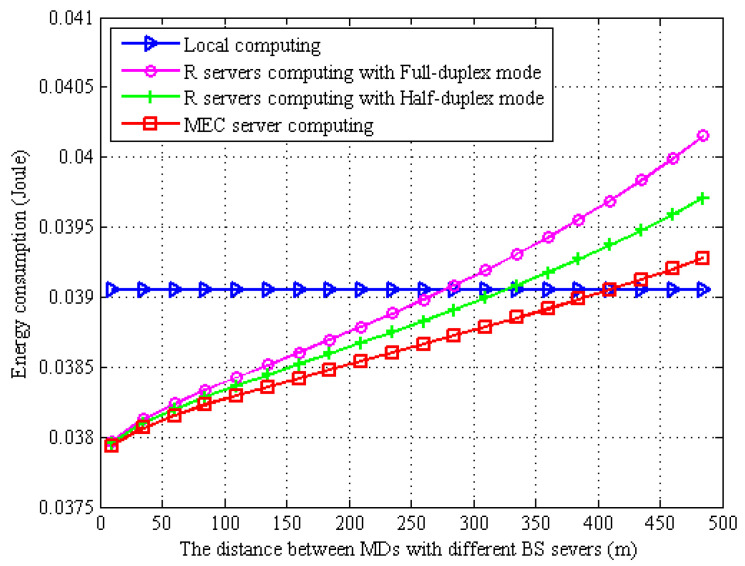
Energy consumption vs. different distance between MDs with BS servers.

In Figure 3, we show the effect of the distance between the MD and the BSs on the energy consumption of MD. As shown in this figure, it can be found that the computational energy consumption of the task does not vary with distance when the MD chooses to compute locally. On the contrary, when the MD chooses to offload to the WPT BS or the MBS for computing, the transmission energy consumption during task offloading increases as the distance between the MDs and the BS increases. When the MD selects full-duplex mode for EH, it will reduce the data transmission rate and thus requires more transmission energy consumption because the full-duplex mode receives and transmits wireless signals at the same time. However, under the half-duplex mode, MDs will harvest energy at first, and then, the task will be offloaded to servers for computing. It can also be found that the MDs that offload to the MBS consume less energy because the channel bandwidth between the MDs and the MBS is larger than the bandwidth between the WPT BS and the MD.

**Figure 4:**
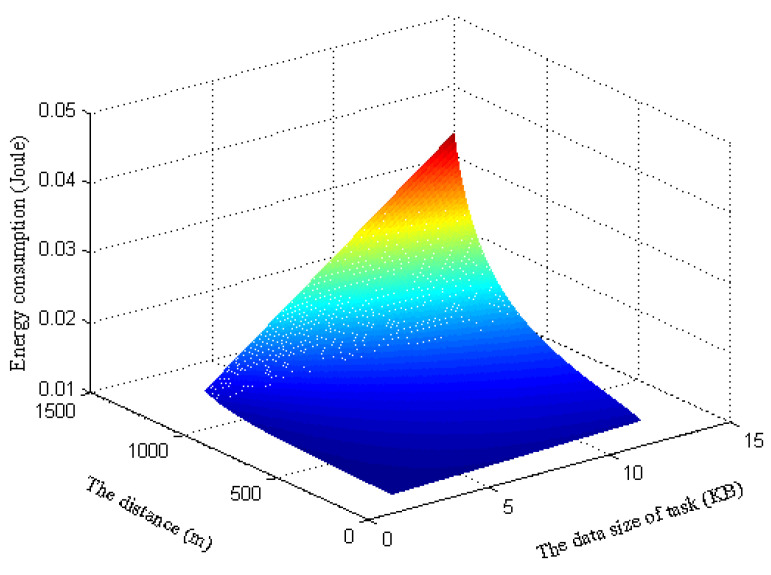
Energy consumption vs. different distances and data sizes of tasks.

Moreover, in Figure 4, we present a three-dimensional diagram of the energy consumption by changing the distance between the MDs and WPT BS as well as the task data size. Such a phenomenon indicates that the energy consumption of the computation task becomes larger as the distance between the MD and the WPT BS increases. Meanwhile, as the data size of the computation task increases, the energy consumption for computation task offloading and task processing tends to increase.

**Figure 5:**
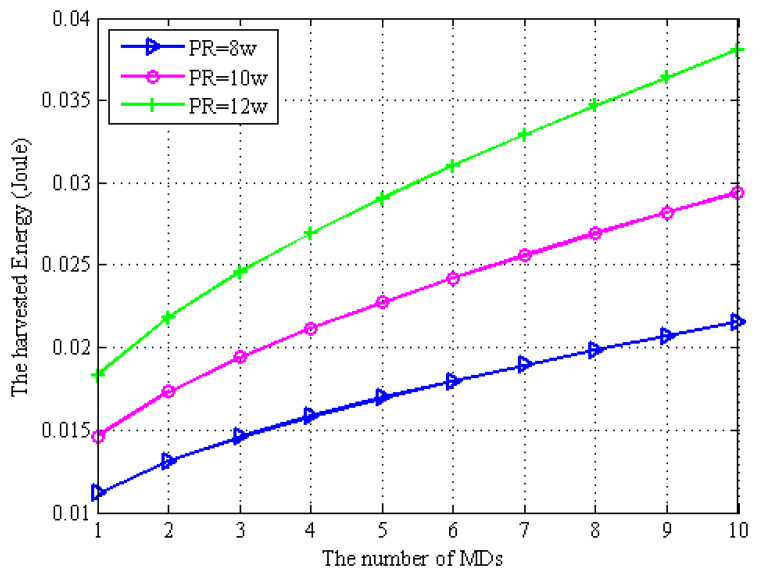
Energy consumption vs. the number of MDs.

**Figure 6:**
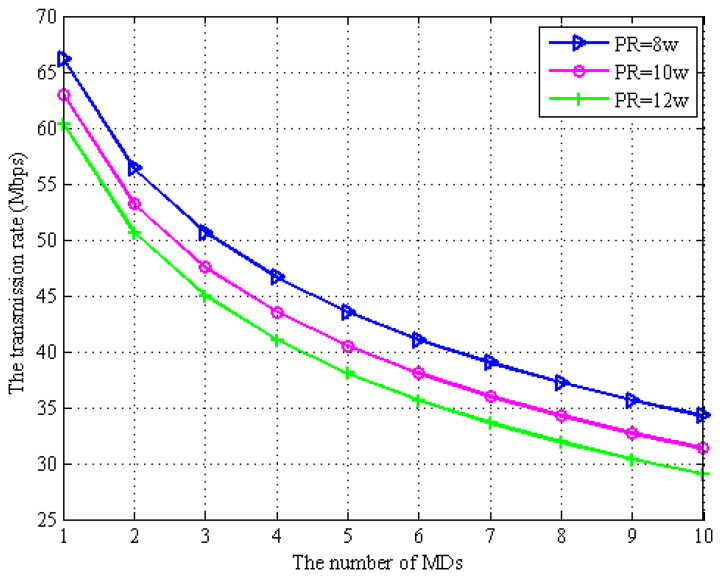
Energy consumption vs. the number of MDs.

In Figure 5 and Figure 6, we describe the transmission rate of computation task offloading data and the EH by MD regarding the number of MDs in the network system proposed in the paper under the full-duplex EH technique based on the MD. We assume that the transmission power of the WPT BS is $P_{R}=\left\{8w,10w,12w\right\}$. From Figure 5, we can observe that as the number of MDs increases, the task offloading rate between the MDs and the WPT BS decreases subsequently. This is because under the full-duplex EH model, self-interference is easily generated, which increases the interference of computation task offloading in the wireless channel and thus decreases the rate of data transmission. It can be seen from Figure 6 that as the number of MDs increases, the offloading transmission rate decreases, which increases the offloading time. In order to complete the task within the maximum allowed delay, the MDs need to harvest more energy provided by the WPT BS. It can be seen from both Figure 5 and Figure 6 that in order to increase the offloading rate, the transmission power between the MDs and the WPT BS needs to be increased.

**Figure 7:**
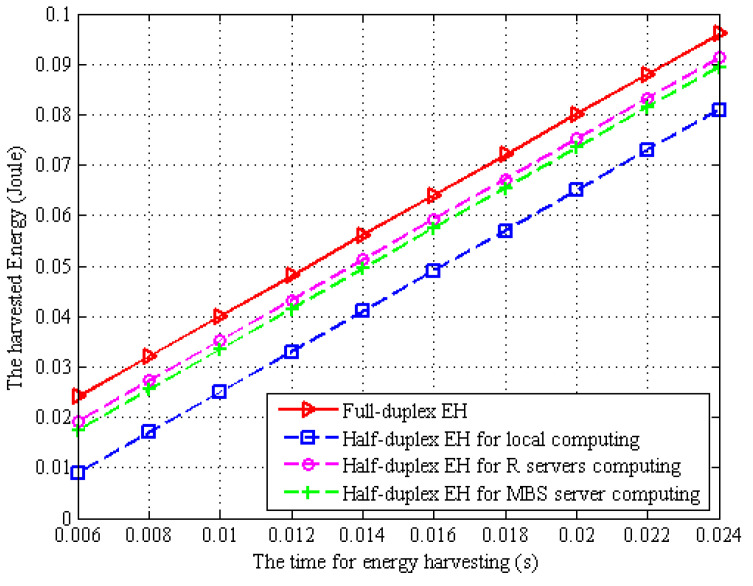
Energy consumption v.s. the time for EH.

In Figure 7, we plot the harvested energy of the MDs based on full-duplex and half-duplex mode. From Figure 7, we can see that the harvested energy by the MDs in full-duplex and half-duplex modes increases linearly with the increment of time. In full-duplex mode, the MDs can perform task offloading at the same time or perform EH during local computation, while in half-duplex mode, the MDs performs EH before task offloading and computation, which will reduce the time for EH, so more energy is harvested in full-duplex mode than in half-duplex mode. In the half-duplex mode, when the MD adopts local computing, the time for task processing increases due to the limited computing capacity of MD, thus reducing the time for EH. On the contrary, when the MDs choose to offload to the WPT BS and MBS server for task computation, the MBS server has a powerful task processing capacity and the time for offloading and computation is reduced; then, the time for EH will increase, and more energy can be obtained.

## 6. Conclusions

In this paper, we have considered a three-tier heterogeneous MEC based on an intelligent network system for offloading energy harvesting. The system model consists of MDs, WPT BS, and MBS integrated with powerful servers, where tasks generated by the user’s MD can be computed locally or offloaded to the wireless energy supply base station servers and the MBS server for computation. In this work, the full/half-duplex energy harvesting-based technology is adopted. To minimize the overall energy consumption for task offloading and computation in heterogeneous network systems, the computation task offloading decision, the energy-harvesting time in full/half-duplex mode, and the energy-harvesting mode selection decision for full/half-duplex is optimized. We show that this optimization problem is a non-convex separable QCQP, which is an NP-hard problem, and the SDR method and the binary recovery method are employed to obtain the optimal decisions while solving for the optimal time resource allocation in different energy-harvesting modes. Simulation results showed that the proposed method and algorithm can achieves the optimal solution. Furthermore, the joint dynamic computation offloading of the computation task and mobility of MDs which based on WPT-based MEC network system is one of the interest for future research.

## Figures and Tables

**Table 1:** Summary of the key notations.

Notations	Meanings
U	The set of MDs in the system
$A_{i}$	The set of offloading decision of MD *i*
*R*	The representation of WPT BS
$F_{i}^{l}$	The computing capacity of MD *i*
$F^{R}$	The computing capacity of WPT BS
$F^{M}$	The computing capacity of MBS server
$d_{i}$	The data size of MD’s computing task
*w*	Represents the number of CPU cycles required to calculate each bit of data
$a_{i,l}$	The decision for local computing of MD *i*
$a_{i,R}$	Implies the MD *i* to select the server of WPT BS to calculate
$a_{i,M}$	Denotes that the MD *i* selects the MBS server for execution
ω	The number of effective capacitors related to chip structure
σ	The path loss factor
$p_{i,R}$	The transmitted power of the MD *i*
$g_{i,R}$	The wireless channel transmission gain between MD *i* and WPT BS
$B_{i,R}$	The wireless transmission bandwidth between MD *i* and WPT BS
$D_{i}^{R}$	The distance between MD *i* and WPT BS
$D_{i,M}$	The distance between the MD *i* and the MBS
$g_{i,M}$	Gain of wireless channel transmission between the MD *i* and MBS
$B_{i,M}$	The wireless transmission bandwidth between MD with the MBS
$p_{i,M}$	Represents the wireless transmission power of the MD *i* to MBS
φ	Represents the amount of energy consumed per CPU cycle by the MBS server to perform computing tasks for MDs
$P_{R}$	The power of energy transmission
$t_{i}$	The time slot
$\tau_{i}$	The time of EH in half-duplex mode
ς	The efficient of EH
*I*	The Gaussian noise

## Data Availability

Not applicable.

## References

[1] Mao Y., You C., Zhang J., Huang K., Letaief K.B. (2017). A Survey on Mobile Edge Computing: The Communication Perspective. IEEE Commun. Surv. Tutor..

[2] Liu L., Chang Z., Guo X., Mao S., Ristaniemi T. (2018). Multi-objective Optimization for Computation Offloading in Fog Computing. IEEE Internet Things J..

[3] Huang T., Lin W., Hong X., Wang X., Wu Q., Li R., Hsu C.-H., Zomaya A.Y. (2022). Adaptive Processor Frequency Adjustment for Mobile-Edge Computing with Intermittent Energy Supply. IEEE Internet Things J..

[4] Han J., Lee G.H., Park S., Choi J.K. (2022). Joint Subcarrier and Transmission Power Allocation in OFDMA-based WPT System for Mobile Edge Computing in IoT Environment. IEEE Internet Things J..

[5] Guo Y., Zhao R., Lai S., Fan L., Lei X., Karagiannidis G.K. (2022). Distributed Machine Learning for Multiuser Mobile Edge Computing Systems. IEEE J. Sel. Top. Signal Process..

[6] Zhao F., Chen Y., Zhang Y., Liu Z., Chen X. (2021). Dynamic Offloading and Resource Scheduling for Mobile-Edge Computing with Energy Harvesting Devices. IEEE Trans. Netw. Serv. Manag..

[7] Chen Y., Zhao F., Chen X., Wu Y. (2022). Efficient Multi-Vehicle Task Offloading for Mobile Edge Computing in 6G Networks. IEEE Trans. Veh. Technol..

[8] Chen J., Chang Z., Guo X., Li R., Han Z., Hämäläinen T. (2021). Resource Allocation and Computation Offloading for Multi-Access Edge Computing With Fronthaul and Backhaul Constraints. IEEE Trans. Veh. Technol..

[9] Chang Z., Gong J., Li Y., Zhou Z., Ristaniemi T., Shi G., Han Z., Niu Z. (2016). Energy Efficient Resource Allocation for Wireless Power Transfer Enabled Collaborative Mobile Clouds. IEEE J. Sel. Areas Commun..

[10] Sun M., Xu X., Huang Y., Wu Q., Tao X., Zhang P. (2021). Resource Management for Computation Offloading in D2D-Aided Wireless Powered Mobile-Edge Computing Networks. IEEE Internet Things J..

[11] Boyd S., Vandenberghe L. (2004). Convex Optimization.

[12] Luo Z.Q., Ma W.K., So M.C., Ye Y., Zhang S. (2010). Semidefinite Relaxation of Quadratic Optimization Problems. IEEE Signal Process. Mag..

[13] Mach P., Becvar Z. (2017). Mobile Edge Computing: A Survey on Architecture and Computation Offloading. IEEE Commun. Surv. Tutor..

[14] Nath S., Wu J. (2020). Deep reinforcement learning for dynamic computation offloading and resource allocation in cache-assisted mobile edge computing systems. Intell. Converg. Netw..

[15] Guo W., Chang Z., Guo X., Wu P., Han Z. (2022). Incentive Mechanism for Edge Computing-based Blockchain: A Sequential Game Approach. IEEE Trans. Ind. Inform..

[16] Du J., Cheng W., Lu G., Cao H., Chu X., Zhang Z., Wang J. (2022). Resource Pricing and Allocation in MEC Enabled Blockchain Systems: An A3C Deep Reinforcement Learning Approach. IEEE Trans. Netw. Sci. Eng..

[17] Xu X., Jiang Q., Zhang P., Cao X., Khosravi M.R., Alex L.T., Qi L., Dou W. (2022). Game Theory for Distributed IoV Task Offloading with Fuzzy Neural Network in Edge Computing. IEEE Trans. Fuzzy Syst..

[18] Zhang J., Du J., Shen Y., Wang J. (2020). Dynamic Computation Offloading with Energy Harvesting Devices: A Hybrid Decision Based Deep Reinforcement Learning Approach. IEEE Internet Things J..

[19] Huang L., Bi S., Zhang Y.J.A. (2020). Deep Reinforcement Learning for Online Computation Offloading in Wireless Powered Mobile-Edge Computing Networks. IEEE Trans. Mob. Comput..

[20] Zhou F., Hu R.Q. (2020). Computation Efficiency Maximization in Wireless-Powered Mobile Edge Computing Networks. IEEE Trans. Wirel. Commun..

[21] Liu Y., Xie S., Yang Q., Zhang Y. (2020). Joint Computation Offloading and Demand Response Management in Mobile Edge Network with Renewable Energy Sources. IEEE Trans. Veh. Technol..

[22] Hu H., Wang Q., Hu R.Q., Zhu H. (2021). Mobility-Aware Offloading and Resource Allocation in a MEC-Enabled IoT Network with Energy Harvesting. IEEE Internet Things J..

[23] Chang Z., Lei L., Zhang H., Ristaniemi T., Chatzinotas S., Ottersten B., Han Z. (2018). Energy-Efficient and Secure Resource Allocation for Multiple-Antenna NOMA with Wireless Power Transfer. IEEE Trans. Green Commun. Netw..

[24] Chang Z., Wang Z., Guo X., Yang C., Han Z., Ristaniemi T. (2019). Distributed Resource Allocation for Energy Efficiency in OFDMA Multicell Networks with Wireless Power Transfer. IEEE J. Sel. Areas Commun..

[25] Wang X., Ning Z., Guo L., Guo S., Gao X., Wang G. (2022). Online Learning for Distributed Computation Offloading in Wireless Powered Mobile Edge Computing Networks. IEEE Trans. Parallel Distrib. Syst..

[26] Liu L., Chang Z., Guo X. (2018). Socially Aware Dynamic Computation Offloading Scheme for Fog Computing System with Energy Harvesting Devices. IEEE Internet Things J..

[27] Liu X., Hu S., Li M., Lai B. (2021). Energy-Efficient Resource Allocation for Cognitive Industrial Internet of Things With Wireless Energy Harvesting. IEEE Trans. Ind. Inform..

[28] Pei X., Duan W., Wen M., Wu Y.-C., Yu H., Monteiro V. (2021). Socially Aware Joint Resource Allocation and Computation Offloading in NOMA-Aided Energy-Harvesting Massive IoT. IEEE Internet Things J..

[29] Lee H., Choi J., Hong D. (2022). Resource Configuration for Full-Duplex-Aided Multiple-Access Edge Computation Offloading. IEEE Trans. Wirel. Commun..

[30] Yang Z., Xu W., Shikh-Bahaei M. (2020). Energy Efficient UAV Communication with Energy Harvesting. IEEE Trans. Veh. Technol..

[31] Tang X., Cai Y., Deng Y., Huang Y., Yang W., Yang W. (2019). Energy-Constrained SWIPT Networks: Enhancing Physical Layer Security with FD Self-Jamming. IEEE Trans. Inf. Forensics Secur..

[32] Shahsavari S., Shirani F., Khojastepour M.A., Erkip E. (2022). Opportunistic Temporal Fair Mode Selection and User Scheduling in Full-Duplex Systems. IEEE J. Sel. Areas Commun..

[33] Gang Q. What is the limit of energy saving by dynamic voltage scaling?. Proceedings of the IEEE/ACM International Conference on Computer Aided Design (ICCAD 2001), IEEE/ACM Digest of Technical Papers (Cat. No.01CH37281).

[34] Burd T.D., Brodersen R.W. (1996). Processor design for portable systems. J. VLSI Signal Process. Syst. Signal Image Video Technol..

[35] Ju H., Zhang R. (2014). Optimal resource allocation in full-duplex wireless-powered communication network. IEEE Trans. Commun..

[36] Du J., Zhao L., Feng J., Chu X. (2018). Computation Offloading and Resource Allocation in Mixed Fog/Cloud Computing Systems with Min-Max Fairness Guarantee. IEEE Trans. Commun..

[37] Chen M., Liang B., Dong M. (2018). Multi-user Multi-Task Offloading and Resource Allocation in Mobile Cloud Systems. IEEE Trans. Wirel. Commun..

[38] Grant M., Boyd S., Ye Y. CVX: MATLAB Software for Disciplined Convex Programming, version 2.0 beta. http://cvxr.com/cvx/.

[39] Chen M., Dong M., Liang B. Joint offloading decision and resource allocation for mobile cloud with computing access point. Proceedings of the 2016 IEEE International Conference on Acoustics, Speech and Signal Processing (ICASSP).

[40] Dinh T.Q., Tang J., La Q.D., Quek T.Q.S. (2017). Offloading in Mobile Edge Computing: Task Allocation and Computational Frequency Scaling. IEEE Trans. Commun..

